# Ontogenetic Shifts in Mycorrhiza-Mediated Neighborhood Effects Among Multi-Stemmed Species in a Subtropical Forest

**DOI:** 10.3390/plants15121784

**Published:** 2026-06-10

**Authors:** Yunquan Wang, Qi Wu, Yidan Yang, Jianhui Ma, Shuisheng Yu, Xiangcheng Mi, Jianhua Chen, Mingjian Yu

**Affiliations:** 1 State Key Laboratory for Vegetation Structure, Function and Construction, College of Life Sciences, Zhejiang University, Hangzhou 310058, China; yqwang@vip.126.com; 2 The Administration Center of Zhejiang Jiulongshan National Nature Reserve, Lishui 323300, China; schhc@sina.com; 3 College of Life Sciences, Zhejiang Normal University, Jinhua 321004, China; wq330327@foxmail.com (Q.W.); 202020200792@zjnu.edu.cn (Y.Y.); 4 Shanghai Chenshan Botanical Garden, Shanghai 201602, China; jianhma@126.com; 5 State Key Laboratory of Vegetation and Environmental Change, Institute of Botany, The Chinese Academy of Sciences, Beijing 100093, China; mixiangcheng@ibcas.ac.cn

**Keywords:** mycorrhizal type, resprouting, multi-stem strategy, ontogenetic shifts, neighborhood effect, Gutianshan subtropical forest

## Abstract

Although plant–mycorrhizal fungi associations play critical roles in maintaining species diversity within forest communities, the influence of tree ontogeny in mediating these effects on species diversity remains poorly understood. In this study, we integrated tree census data with information on the mycorrhizal types and sprouting ability of multi-stemmed species from a subtropical forest to assess how mycorrhiza-mediated neighborhood interactions affecting survival vary across ontogenetic stages (sapling, juvenile and adult stages) and how these effects correlate with sprouting ability. Our results revealed pervasive ontogenetic shifts in mycorrhiza-mediated neighborhood effects on tree survival for multi-stemmed species. AM heterospecific neighbors consistently exerted positive effects on tree survival across all life stages. In contrast, ErM heterospecific neighbors significantly influenced survival only at the sapling stage, whereas EcM heterospecific neighbors had significant effects during the juvenile and adult stages. When focal individuals were classified by mycorrhizal type, AM focal plants were significantly influenced by three types of mycorrhizal heterospecific neighbors, with the effect of AM heterospecific neighbors at the sapling stage being significantly greater than those of EcM or ErM heterospecific neighbors. Notably, AM heterospecific neighbors were critical predictors of survival for EcM focal plants during both the juvenile and adult stages, while AM and EcM heterospecific neighbors jointly the enhanced survival of ErM focal plants during the adult stage. Moreover, the effects of both AM and EcM heterospecific neighbors increased significantly with the sprouting ability of multi-stemmed species, particularly at the sapling stage. Our study highlights the importance of incorporating tree ontogeny and mycorrhizal symbiosis types into the assessment of factors contributing to species coexistence among long-lived organisms.

## 1. Introduction

Understanding the mechanisms that maintain species diversity in forest communities remains a central challenge in ecology. Beyond abiotic factors such as light and nutrients, biotic processes, particularly negative density dependence [1,2] and mycorrhiza-mediated plant–soil feedbacks [3,4,5,6,7], are increasingly recognized as key drivers of species coexistence. Empirical evidence indicates that ontogenetic shifts in long-lived trees can fundamentally alter competitive dynamics and niche partitioning across life stages [8,9,10]. Yet, whether mycorrhiza-mediated neighborhood effects vary across ontogenetic stages and how such shifts correlate with life-history traits such as sprouting ability remains poorly understood.

Mycorrhizal fungi form mutualistic associations with the majority of forest trees, supplying essential nutritional resources in return for photosynthetically derived carbon from host plants [11,12,13]. Four major types of mycorrhizal associations have been classified based on their structural and functional characteristics: arbuscular mycorrhizal (AM), ectomycorrhizal (EcM), ericoid mycorrhizal (ErM), and orchid mycorrhizal (OrM) fungi [13]. Accumulating evidence indicates that AM and EcM fungi differ significantly in their capacity to modulate negative plant–soil feedbacks [4,5,14]. Tree species associated with EcM fungi generally experience weaker negative plant–soil feedbacks than those associated with AM fungi, leading to a reduction in conspecific negative density dependence in forest communities [4,11]. These differences in feedback modulation ultimately shape the relative abundance and coexistence of tree species in diverse forest communities [3,6].

Mycorrhizal fungi might mediate not only conspecific interactions but also heterospecific interactions among plants sharing the same mycorrhizal association, a phenomenon known as the con-mycorrhizal density dependence [6,15]. Given the relatively low host specificity of AM fungi, AM-associated plant species can obtain facilitative benefits from heterospecific neighbors with compatible mycorrhizal status, thereby enhancing positive plant–plant interactions. In contrast, mycorrhizal fungi might also mediate competitive interactions both among plants and between plants and fungi among host species associated with different mycorrhizal types [3,16], a pattern that theoretically supports the predominance of hetero-mycorrhizal competition over facilitation [6]. Moreover, AM-associated plant species not only benefit from the presence of EcM-associated neighbors but also exhibit higher rates of colonization by EcM fungi compared to certain native EcM host species [4].

While AM and EcM symbioses have received extensive attention, ErM fungi represent a functional distinct guild that remain underexplored in diversity maintenance frameworks. ErM fungi exclusively colonize plants within the Ericaceae family (e.g., *Rhododendron latoucheae*); most species in this family lack root hairs and thus depend entirely on ErM fungi for nutrient acquisition [13]. ErM fungi can release soil nutrients through the decomposing complex and recalcitrant organic substrates, thereby playing a keystone role in mobilizing organic nutrients, particularly in nutrient-poor ecosystems (e.g., rhododendron forests) [17,18]. Despite their ecological importance, ErM-mediated neighborhood interactions are rarely incorporated into frameworks of mutualism-driven species coexistence [19,20], representing a critical gap that this study aims to address.

Integrating mycorrhizal symbioses with key plant life-history strategies, such as regeneration modes, may provide deeper mechanistic insight regarding community assembly. Basal sprouting, the production of multi-stemmed individuals from root collars, represents a widespread life-history strategy in woody plants that enables rapid post-disturbance regeneration and in situ niche persistence [21,22,23]. Sprouting ability varies substantially among species and correlates with demographic performance and community dynamics [24,25,26,27]. Emerging evidence suggests that neighborhood competition may influence sprouting by altering resource allocation between main stems and sprouts [27]. However, whether sprouting ability modulates the strength of mycorrhiza-mediated neighborhood effects, and thereby contributes to community diversity and species coexistence, remains poorly understood.

Chinese subtropical forests, despite their relatively limited global extent [28], harbor exceptional biodiversity and function as major terrestrial carbon sinks [29,30]. The Gutianshan National Nature Reserve in Zhejiang Province protects a representative subtropical evergreen broad-leaved mature forest [31,32]. Tree species within this forest community commonly exhibit prevalent sprouting [26,33,34], providing an ideal system to examine how mycorrhizal-mediated neighborhood effects affect species survival vary across ontogeny and correlate with sprouting ability. Here, we integrated tree census data with mycorrhizal type information from a 5 ha forest plot to address two questions: (1) How do mycorrhiza-mediated neighborhood effects on tree survival vary across tree ontogeny in multi-stemmed species? (2) Do ontogenetic shifts in these neighborhood effects correlate with species-specific sprouting abilities? We hypothesized that if sprouting enables individuals to exploit mycorrhizal-mediated facilitation more effectively by maintaining access to shared hyphal networks during early ontogeny, then species with higher sprouting ability should exhibit stronger positive responses to con-mycorrhizal heterospecific neighbors, particularly at the sapling stage when sprouting is most prevalent and mycorrhizal network dependence is greatest.

## 2. Results

### 2.1. Ontogenetic Shifts in Mycorrhiza-Dependent Neighborhood Effects on Survival

**Figure 1:**
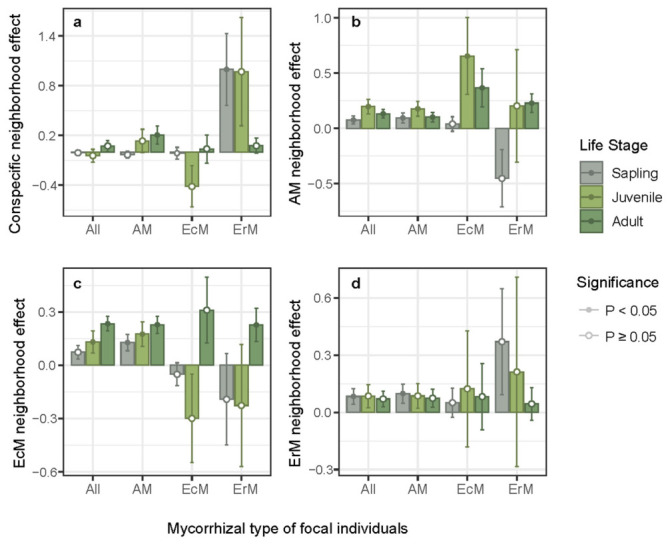
The effects of neighborhood density on tree survival for multi-stemmed species across ontogenetic stages. Bayesian generalized linear mixed model results show posterior median estimates (circles) with ±1 standard error (lines) for the effects of (**a**) conspecific neighbors, (**b**) arbuscular mycorrhizal (AM) heterospecific neighbors, (**c**) ectomycorrhizal (EcM) heterospecific neighbors, and (**d**) ericoid mycorrhizal (ErM) heterospecific neighbors on all AM, EcM, and ErM focal individuals. Filled circles indicate statistically significant effects; open circles indicate non-significant effects. Bar heights represent posterior median effect sizes.

**Figure 2:**
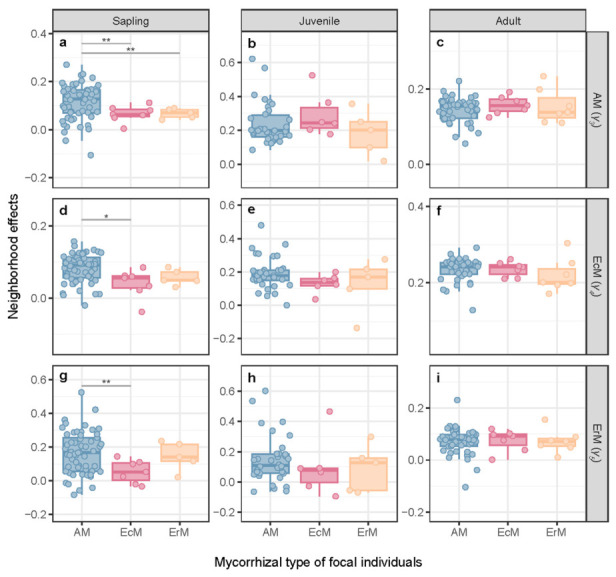
Mycorrhiza-mediated neighborhood effects vary across mycorrhizal types and tree ontogenetic stages. Boxplots show species-level posterior distributions of γ coefficients (γ_3_–γ_5_) derived from general models, representing effects mediated by (**a**–**c**) AM, (**d**–**f**) EcM, and (**g**–**i**) ErM heterospecific neighbors. The center lines represent medians, boxes indicate interquartile ranges (IQRs), and whiskers extend to 1.5 × IQR. Asterisks denote statistically significant pairwise differences among mycorrhizal groups (two-tailed t-tests; * *p* < 0.05, ** *p* < 0.01, and *** *p* < 0.001).

The effects of mycorrhiza-mediated neighborhood interactions on the survival of multi-stemmed trees varied significantly across tree ontogeny (Figure 1 and Figure 2, Table S1). At the community level, AM heterospecific neighbors have a consistent and positive influence on tree survival across all life stages, whereas the conspecific neighborhood effects were not statistically significant (Figure 1). In contrast, the impacts of both ErM and EcM heterospecific neighbors on survival varied with tree ontogeny. Specifically, survival was significantly affected by ErM heterospecific neighbors during the sapling stage, as well as by EcM heterospecific neighbors at both the juvenile and adult stages (Figure 1).

When focal individuals were classified according to their own mycorrhizal type, distinct patterns were found. AM focal plants were significantly influenced by all three mycorrhizal type heterospecific neighbors, although the effect of ErM heterospecific neighbors was restricted to the sapling stage (Figure 1). Moreover, AM heterospecific neighbors had a consistently stronger positive effect on AM focal plants compared to EcM or ErM heterospecific neighbors, with this difference being most evident at the sapling stage (Figure 2a,d,g). In contrast, EcM focal plants were significantly affected by AM heterospecific neighbors during both the juvenile and adult stages (Figure 1b). ErM focal plants showed significant responses only at the adult stage, when survival was positively influenced by both AM and EcM heterospecific neighbors (Figure 1b,c).

### 2.2. Relationship Between Mycorrhiza-Mediated Neighborhood Effects and Species-Specific Sprouting Ability

**Figure 3:**
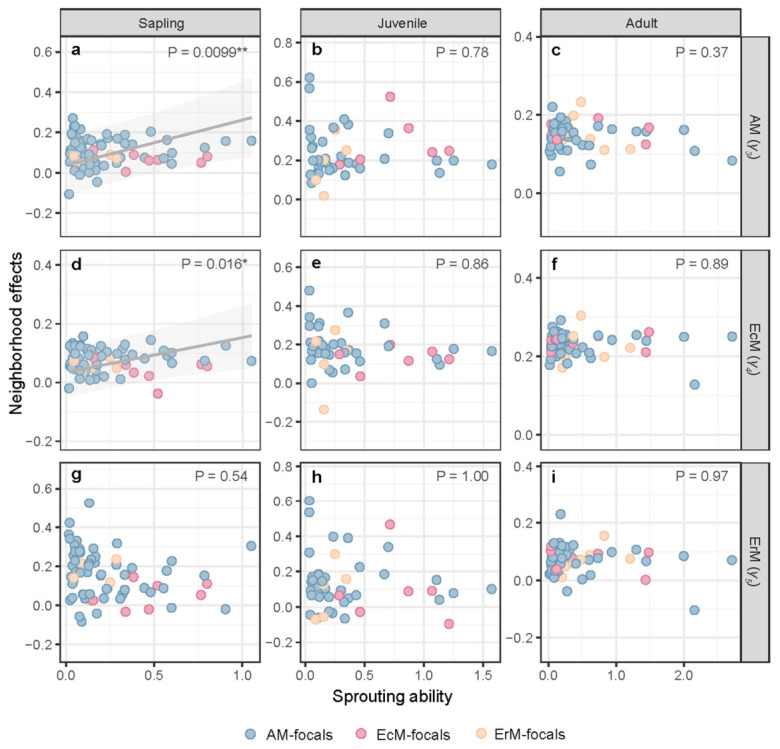
The relationship between mycorrhiza-mediated neighborhood effects and the sprouting ability of multi-stemmed species across tree ontogenetic stages. Solid lines depict predicted relationships from multiple linear regression analyses (shaded bands = 95% confidence intervals, CIs) between the sprout-to-main-stem ratio (RS) and neighborhood effects (i.e., γ coefficients from mycorrhizal-type-specific models), adjusted for species abundance (log-transformed). Points represent species-level posterior distributions of γ coefficients (γ_3_–γ_5_) from mycorrhizal-type-specific models, representing effects mediated by (**a**–**c**) AM, (**d**–**f**) EcM, and (**g**–**i**) ErM heterospecific neighbors. * *p* < 0.05, ** *p* < 0.01, and *** *p* < 0.001.

Multiple linear regression analyses revealed that the relationship between mycorrhiza-mediated neighborhood effects and the sprouting ability of multi-stemmed species is ontogenetic-dependent, with significant correlations observed exclusively during the sapling stage (Figure 3). Specifically, the effects of both AM and EcM heterospecific neighbors increased significantly with greater sprouting ability during the sapling stage (Figure 3, Table S2), although this positive association diminished with increasing species abundance (Table S2). In contrast, no significant correlation was detected between sprouting ability and the effects of ErM heterospecific neighbors during the sapling stage, nor with the effects of AM, EcM, or ErM heterospecific neighbors in later life stages (i.e., juvenile and adult stages, Figure 3, Table S2).

## 3. Discussion

### 3.1. Mycorrhizae-Mediated Neighborhood Effects on Survival Across Tree Ontogeny

We found no significant conspecific negative density dependence (CNDD) across any ontogenetic stage examined, contrasting with widely observed CNDD patterns in (sub)tropical forests [35,36,37]. The absence of CNDD may reflect several non-mutually exclusive mechanisms. First, pathogen pressure in this mid-latitude subtropical forest may be lower than in low-latitude tropical systems, where Janzen–Connell effects are most pronounced [9,38]. Second, facilitative interactions via shared mycorrhizal networks may offset pathogen-driven mortality, particularly when mycorrhizal colonization rates are high [3,4]. Third, our analytical framework, which partitions heterospecific neighbors by mycorrhizal type rather than aggregating them into a single category, enables the detection of mycorrhizal-mediated facilitation that may be masked under conventional conspecific/heterospecific classifications. The absence of CNDD does not indicate the absence of density-dependent processes; rather, it suggests that multiple mechanisms operate simultaneously, with mycorrhizal facilitation potentially attenuating pathogen-driven mortality under specific demographic and environmental conditions.

AM heterospecific neighborhood effects exhibited consistent positive effects across all stages, whereas EcM and ErM heterospecific neighborhood effects emerged predominantly in later and earlier stages, respectively. The ontogenetically pervasive facilitation by AM neighbors likely reflects two mechanisms: (1) the dominance of AM-associated species increases encounter probabilities for shared network formation, and (2) AM fungi’s rapid hyphal turnover and extensive extraradical networks enable efficient resource redistribution [3,4,6]. In contrast to the persistent facilitative effects observed in AM-mediated neighborhood effects, EcM species are likely to immobilize nutrients to maintain mycelial integrity, thereby reducing soil nutrient availability [39]. As a result, the benefits conferred through common mycorrhizal networks such as nutrient transfer may be masked during early ontogeny [40]. Conversely, ErM species may suppress pathogen activity by limiting mineral nutrient availability [41], but their reduced influence in later developmental stages aligns with declining plant sensitivity to such protective effects as individuals mature [42].

The mycorrhizal identity of focal species modulated neighborhood effect patterns. AM-associated focal species received consistent facilitation from both AM and EcM heterospecific neighbors across all life stages, whereas the effects of ErM heterospecific neighbors were limited to the sapling stage. Notably, AM sapling derived greater benefits from EcM and ErM heterospecific neighbors compared to EcM- or ErM-associated species. These findings extend previous findings of cross-mycorrhizal facilitation [6] and may reflect the fact that AM-associated species can access nutrients mobilized by EcM exoenzymes without bearing the full carbon costs of EcM colonization [4]. If these facilitative asymmetries persist across demographic transitions, such dynamics may drive directional succession toward AM-dominated communities in the Gutianshan forest, contrasting with the current state of AM-EcM codominance. Given the greater prevalence and frequent monodominance of EcM-associated trees in temperate forests [43,44], the divergent mycorrhizal composition suggests that temperate forests may yield opposing patterns of neighborhood interaction (i.e., greater benefits for EcM species through mycorrhizae-mediated facilitation relative to other mycorrhizal groups). Therefore, further studies are critically needed to address the current lack of cross-biome comparative data on mycorrhiza-mediated neighborhood interactions.

EcM-associated focal species exhibited responsiveness to AM heterospecific neighbors exclusively in later life stages. As EcM trees transition from light-limited saplings to nutrient-limited later stages [45], the functional value of accessing AM-mobilized nutrients increases, potentially explaining the stage-specific facilitation. In contrast, ErM-associated focal species benefited from hetero-mycorrhizal neighbors without significant responses to con-mycorrhizal effects, a pattern consistent with mycorrhizal niche complementarity; ErM specialists occupy narrow functional spaces due to high host specificity [13], yet they can access nutrients mobilized by the spatially extensive AM/EcM networks that dominate the community [4]. However, such hetero-mycorrhizal facilitation appears insufficient to promote ErM dominance, unlike patterns observed in certain temperate forests [19,20]. A previous study posits that mature EcM trees facilitate both AM- and EcM-associated species to stabilize coexistence [46]. However, our findings indicate that AM-mediated facilitation plays a disproportionately strong role in structuring subtropical forest demography. Nevertheless, its relative importance compared to other coexistence mechanisms (e.g., niche partitioning, storage effects) requires multivariate demographic modeling.

### 3.2. Ontogenetic Shifts in Mycorrhiza-Mediated Neighborhood Effects Correlate with the Sprouting Ability of Multi-Stemmed Species

Our findings demonstrate a significant positive correlation between sprouting ability and the magnitude of AM/EcM-mediated neighborhood facilitation, specifically at the sapling stage. This sprouting–facilitation relationship supports our hypothesis that species with greater sprouting ability are more likely to benefit from heterospecific neighbor facilitation at earlier stages. Species with greater sprouting abilities tend to develop thicker root diameters [47], a morphological adaptation that enhances the potential for mycorrhizal colonization [48,49]. The observed pattern in AM-associated species further aligns with successional trajectory toward AM-dominated assemblages. However, we emphasize that current evidence remains indirect, relying on categorical comparisons (sprouting vs. non-sprouting species) rather than continuous trait analyses [47]. Future studies should explicitly quantify how interspecific variation in sprouting ability modulates root functional traits and the outcomes of mutualistic interactions.

Intriguingly, facilitative effects diminished with increasing species abundance, indicating that rare sprouting species derive greater benefits from AM- and EcM-associated heterospecific neighbors compared to common species with equivalent sprouting ability. This pattern suggests that mycorrhizal facilitation may provide density-dependent fitness advantages that disproportionately benefit rare species, potentially contributing to negative frequency-dependent selection that stabilizes diversity [50].

An alternative mechanism may counterbalance sprouting–facilitation interactions at the sapling stage. Prolific sprouters typically prioritize horizontal clonal expansion over vertical growth [47], restricting these species to low-light understory environments where the carbon costs of maintaining mycorrhizal symbiosis may outweigh their benefits [51]. When this constraint is mitigated by root traits that enhance colonization potential (e.g., thicker root diameters in strong sprouters), neutral associations emerge, as exemplified by ErM-mediated effects. ErM fungi, which function as facultative saprotrophs capable of decomposing organic litter without obligatory root colonization [52], decouple symbiosis outcomes from sprouting ability.

Our results demonstrate a decoupling between mycorrhiza-mediated facilitation and sprouting ability beyond the sapling stage. Both mycorrhizal dependency and sprouting ability decline with tree ontogeny [21,53], suggesting that late-stage species reduce their reliance on regeneration-related traits and mycorrhizal facilitation, factors that are critical during early establishment. Concurrently, as species mature, resource limitations shift from light to soil nutrients and water [45], thereby diminishing the significance of the sprouting–height trade-off. These dynamics may explain the prevalence of resprouting in disturbed communities, where early-stage sprouters leverage mycorrhizal feedbacks to achieve rapid regeneration [21,54]. Collectively, these findings indicate that sapling-stage sprouting ability emerges as a pivotal regeneration filter, regulating access to mycorrhizal mutualisms that underpin establishment success and long-term coexistence in dynamic forest ecosystems.

## 4. Materials and Methods

### 4.1. Study Site and Tree Census

This study was conducted in a predominantly old-growth subtropical forest within the 5 ha forest dynamics plot (29.25° N, 118.12° E) located in the Gutianshan National Nature Reserve, Zhejiang Province, eastern China [31]. This region has a mid-subtropical monsoon climate, with a mean annual temperature of 15.3 °C and a mean annual precipitation of 1964 mm [36]. Soils in the reserve are derived from granite weathering and are classified as red, red-yellow, yellow-red, and marsh types [55]. The vegetation is primarily composed of evergreen broad-leaved forests, which are widely distributed in subtropical lowland areas below 800 m above sea level. The dominant canopy species are *Castanopsis eyrei* (Fagaceae, EcM) and *Schima superba* (Theaceae, AM) in the reserve [32,55].

The 5 ha forest dynamics plot was established in 2002 following the standards of the CTFS-ForestGEO protocol [56]. The plot extends 200 m in the east–west direction and 250 m in the north–south direction, featuring hillsides on the northern and southern edges and a central valley. The cross-sectional profile of the terrain resembles an irregular “V” shape [31,57]. All freestanding woody stems with DBH (diameter at breast height, 1.3 m) ≥ 1 cm in the plot were tagged, mapped, identified to species, and measured for DBH, number of sprouts, and number of branches [31]. Survival was assessed at each five-year interval, and new recruits were tagged, measured, mapped, and identified. Species exhibiting root sprouting (i.e., multi-stemmed species) are highly prevalent within this forest community [26], accounting for 73.4% of species richness in the plot. Additional foundational information regarding the study site, establishment procedures, and investment details is provided in Jiang et al. [31].

### 4.2. Tree Ontogenetic Classes and Mycorrhizal Type

To account for ontogenetic differences among growth forms, species were classified as shrubs (maximum height < 5 m) and trees (maximum height ≥ 5 m) following the approach of Zheng et al. [58]. Life stage thresholds were defined as follows: for shrubs, saplings (DBH 1–1.5 cm), juveniles (1.5–2 cm), and adults (≥2 cm); for trees, saplings (1–5 cm), juveniles (5–10 cm), and adults (≥10 cm) [59]. Additionally, following established methodologies [60,61], species were categorized by mycorrhizal type into arbuscular mycorrhizal (AM), ectomycorrhizal (EcM), or ericoid mycorrhizal (ErM) based on established mycorrhizal databases (Table 1). The single non-mycorrhizal species, *Cladrastis wilsonii* (Fabaceae), was excluded from subsequent analysis.

### 4.3. Sprouting Ability of Multi-Stemmed Species

Sprouting ability for each multi-stemmed species was quantified as the mean root-sprout-to-main-stem ratio, calculated by dividing the total number of basal sprouts by the total number of main stems across all conspecific individuals following established methodologies [26,62,63]. Based on tree census data collected in the plot from 2012 to 2017, we first assessed whether individuals exhibited sprouting regeneration. This enabled us to distinguish multi-stemmed individuals (i.e., those with one or more root sprouts attached to a main stem) from single-stemmed individuals.

### 4.4. Neighborhood Variables

For each focal tree individual, surrounding trees were classified into four groups based on mycorrhizal type, and corresponding neighborhood indices were computed to reflect the effects of conspecific neighbors, con-mycorrhizal heterospecific neighbors, and hetero-mycorrhizal heterospecific neighbors on focal individuals with specific mycorrhizal types. These indices included the conspecific neighbor index (CI), the heterospecific AM neighbor index (HI-AM), the heterospecific EcM neighbor index (HI-EcM), and the heterospecific ErM neighbor index (HI-ErM) [4]. Each index incorporates both the basal area of neighboring trees and their distance to the focal individual [64,65]. For clarity and brevity, only the formula for CI (m^2^/m) is presented below:

(1) $$\begin{array}{c} CI\;=\;\sum_{i}^{N}\frac{{\mathrm{BA}}_{i}}{{\mathrm{Distance}}_{i}} \end{array}$$

where *N* represents the neighbors within 10 m radius, *BA_i_* is the basal area of the neighboring individual *i*, and *Distance_i_* is the distance between neighbor *i* and the focal individual. Focal individuals were restricted to multi-stemmed species located at least 10 m from plot edges and required to have at least five 5 representative individuals per life stage [35], resulting in a final dataset of 15,083 individuals from 94 species. We selected a 10 m radius based on previous evidence indicating that trees interact most intensively with their larger adult neighbors in this spatial scale [4,65].

### 4.5. Statistical Analysis

Bayesian generalized linear mixed models (BGLMMs) were employed to assess the effects of mycorrhiza-mediated neighborhood composition, specifically the conspecific density and heterospecific densities of AM, EcM, and ErM neighbors, on species survival across each life stage. A two-level regression structure was developed.

In the community-level regression, we modeled the probability of individual survival *S* for individual *k* of species *j* in plot *i* as a function of DBH and neighborhood variables for each life stage:

(2) $$S_{\mathrm{ijk}}\;\sim \;\mathrm{Bernoulli}\;(p_{\mathrm{ijk}})$$

(3) $$\begin{array}{c} logit\;(p_{\mathrm{ijk}})=\beta_{0}+\beta_{1}\times ln\;({\mathrm{DBH}}_{\mathrm{ijk}})+\beta_{2}\times {\mathrm{Con}}_{\mathrm{ijk}}+{\;\beta }_{3}\times {\mathrm{AM}}_{\mathrm{ijk}}\\ +\beta_{4}\times {\mathrm{EcM}}_{\mathrm{ijk}}+\beta_{5}\times {\mathrm{ErM}}_{\mathrm{ijk}}+\varphi_{i}+\varphi_{j} \end{array}$$

where *Con*, *AM*, *EcM*, and *ErM* represent the densities of conspecific and heterospecific AM, EcM, and ErM tree neighbors, with random intercepts for plot (*φ_i_*) and species (*φ_j_*). We fitted general models using all individuals of sprouting species collectively to evaluate the effects of conspecific neighbors and heterospecific neighbors with specific mycorrhizal types on the survival of focal individuals at the community level. Additionally, we fitted mycorrhizal-type-specific models separately for individuals sharing the same mycorrhizal type to assess the effects of conspecific neighbors, con-mycorrhizal heterospecific neighbors, and hetero-mycorrhizal heterospecific neighbors on the survival of focal individuals with specific mycorrhizal types.

In the species-level regression, we incorporated species as random slopes for each neighborhood variable separately into our general models:

(4) $$\begin{array}{c} logit\;(p_{\mathrm{ijk}})=\beta_{0}+\beta_{1}\times ln\;({\mathrm{DBH}}_{\mathrm{ijk}})+\beta_{2}\times {\mathrm{Con}}_{\mathrm{ijk}}+{\;\gamma }_{3j}\times {\mathrm{AM}}_{\mathrm{ijk}}\\ +\gamma_{4j}\times {\mathrm{EcM}}_{\mathrm{ijk}}+\gamma_{5j}\times {\mathrm{ErM}}_{\mathrm{ijk}}+\varphi_{i}+\varphi_{j} \end{array}$$

where *γ*_3*j*_, *γ*_4*j*_, and *γ*_5*j*_ represent the strength of neighborhood effects experienced by species *j* that are mediated by AM, EcM, and ErM heterospecific trees, respectively. We used pairwise t-tests to examine whether the strength of neighborhood effects differed significantly between species with different mycorrhizal types.

Multiple linear regression models (MLRMs) were employed to examine the relationship between sprouting ability and the strength of neighborhood effects. Log-transformed species abundance and its interaction with sprouting ability were included as covariates to control for potential confounding effects arising from abundance-related processes [66,67]. Comparative analyses across mycorrhizal types were based on neighborhood effect estimates derived from mycorrhizal-type-specific models. We employed t-tests to compare the magnitude of mycorrhiza-mediated neighborhood effects between species of different mycorrhizal types within each ontogenetic stage.

All statistical analyses and visualizations were conducted in R version 4.3.1 (http://www.R-project.org/). Explanatory variables were standardized by subtracting the mean and dividing by one standard deviation prior to modeling. BGLMMs were fitted using the *brms* package [68]. Bayesian models were specified with normal priors for coefficients and were fitted with five Markov chains (2000 iterations per chain, 1000 warm-up iterations) using a seed value of 2024 to ensure reproducibility.

## 5. Conclusions

This study uncovers that tree survival depends on ontogenetic stage and the mycorrhizal types of both the focal tree and its neighboring trees in subtropical forests dominated by multi-stemmed species. AM heterospecific neighbors positively influenced survival throughout all stages. However, ErM neighbors affected survival mainly during the sapling stage, while EcM neighbors had significant impacts on the survival of focal multi-stemmed species during the juvenile and adult stages. AM plants were influenced by all three heterospecific neighbors associated with three mycorrhizal types, with AM neighbors having the strongest effect during the sapling stage. AM heterospecific neighbors were key predictors for the survival of focal EcM species in later stages, and AM and EcM heterospecific neighbors together enhanced the survival of focal ErM species in the adult stage. Additionally, the positive effects of AM and EcM heterospecific neighbors on survival increased with sprouting ability at the sapling stage. Overall, our results demonstrate that the effects of mycorrhiza-mediated heterospecific neighborhood interactions on the survival of multi-stemmed species vary significantly across tree ontogenetic stages and mycorrhizal types. Furthermore, these effects exhibit a significant correlation with species’ sprouting abilities exclusively during the sapling stage. Our study underscores the importance of integrating tree ontogeny and mycorrhizal types into assessments of factors contributing to species coexistence among long-lived organisms.

## Figures and Tables

**Table 1:** The number of focal multi-stemmed species by mycorrhizal type and ontogenetic stage.

Mycorrhizal Type	Sapling	Juvenile	Adult
AM	71	43	51
EcM	7	7	7
ErM	6	6	7

## Data Availability

The original contributions presented in this study are included in the article/Supplementary Materials. Further inquiries can be directed to the corresponding authors.

## Supplementary Materials

The following supporting information can be downloaded at https://www.mdpi.com/article/10.3390/plants15121784/s1. Table S1. The parameter estimates and 95% confidence intervals (95% CI) for individual-level general and mycorrhizal models with random intercepts are shown. Table S2. The parameter estimates and standard error (SE) for the relationship between the strength of neighborhood effects and sprouting ability (Sprout/main stem ratio), log-transformed abundance and their interaction terms for each species are shown.
