# Supplementary material for: Ontogenetic Shifts in Mycorrhiza-Mediated Neighborhood Effects Among Multi-Stemmed Species in a Subtropical Forest
Source: Plants (Basel). 2026 Jun 10;15(12):1784. doi: 10.3390/plants15121784 (PMC13307523; doi:10.3390/plants15121784)
Supplement: Supplementary file 1 [file plants-15-01784-s001.zip › plants-4354569-supplementary.pdf]

Supporting Information

Table S1. The parameter estimates and 95% confidence intervals (95% CI) for individual-level general and mycorrhizal models with random intercepts are shown. Non-zero confidence intervals indicate significant effects on survival and are highlighted in bold. Log (DBH), the log-transformed diameter at breast height (DBH); Con, conspecific neighbor density; AM, EcM, and ErM, the density of heterospecific AM, EcM, and ErM tree neighbors, respectively.

Life stage	Parameter estimates (95% CI)				
	Log (DBH)	Con	AM	EcM	ErM
General models					
All	**0.5787**<br>**(0.5221 – 0.6346)**	0.0262<br>(-0.0317 – 0.0851)	**0.1027**<br>**(0.0559 – 0.1494)**	**0.1296**<br>**(0.0782 – 0.1815)**	**0.0757**<br>**(0.0216 – 0.1300)**
Sapling	**0.2229**<br>**(0.1493 – 0.2979)**	-0.0092<br>(-0.764 – 0.0577)	**0.0757**<br>**(0.0051 – 0.1485)**	0.0732<br>(-0.0004 – 0.1475)	**0.0834**<br>**(0.0026 – 0.1640)**
Juvenile	**0.8729**<br>**(0.6198 – 1.1379)**	-0.0441<br>(-0.1843 – 0.1139)	**0.1979**<br>**(0.0716 – 0.3280)**	**0.1311**<br>**(0.0112 – 0.2513)**	0.0854<br>(-0.0325 – 0.2063)
Adult	**0.6278**	0.0729	**0.1324**	**0.2337**	0.0701

	**(0.4921 – 0.7660)**	(-0.0471 – 0.2099)	**(0.0596 – 0.2084)**	**(0.1549 – 0.3186)**	(-0.0073 – 0.1523)
Mycorrhizal models (AM species)					
All	**0.5145**	0.0646	**0.1053**	**0.1645**	**0.0847**
	**(0.4466 – 0.5843)**	(-0.0213 – 0.1616)	**(0.0495 – 0.1626)**	**(0.1047 – 0.2260)**	**(0.0210 – 0.1513)**
Sapling	**0.3723**	-0.0334	**0.0956**	**0.1276**	**0.0988**
	**(0.2752 – 0.4692)**	(-0.1114 – 0.0506)	**(0.0086 – 0.1841)**	**(0.0380 – 0.2190)**	**(0.0031 – 0.1986)**
Juvenile	**0.8894**	0.1330	**0.1777**	**0.1749**	0.0859
	**(0.5745 – 1.1972)**	(-0.1192 – 0.4353)	**(0.0485 – 0.3118)**	**(0.0436 – 0.3118)**	(-0.0417 – 0.2131)
Adult	**0.5828**	**0.2054**	**0.1025**	**0.2281**	0.0740
	**(0.4130 – 0.7533)**	**(0.0124 – 0.4347)**	**(0.0185 – 0.1891)**	**(0.1339 – 0.3224)**	(-0.0183 – 0.1681)
Mycorrhizal models (EcM species)					
All	**0.8366**	-0.0221	0.0882	-0.0269	0.0595
	**(0.7076 – 0.9705)**	(-0.1518 – 0.1153)	(-0.0229 – 0.1995)	(-0.1375 – 0.0836)	(-0.0733 – 0.1971)
Sapling	-0.0030	-0.0139	0.0403	-0.0513	0.0506
	(-0.1190 – 0.1139)	(-0.1527 – 0.1229)	(-0.0874 – 0.1710)	(-0.1776 – 0.0765)	(-0.0945 – 0.2021)

Juvenile	0.2944<br>(-0.2105 – 0.8081)	-0.4158<br>(-0.9479 – 0.0259)	**0.6541**<br>**(0.0462 – 1.4099)**	-0.2993<br>(-0.7927 – 0.1832)	0.1236<br>(-0.4413 – 0.7516)
Adult	**0.5641**<br>**(0.2147 – 0.8979)**	0.0377<br>(-0.2800 – 0.3851)	**0.3666**<br>**(0.0418 – 0.7187)**	0.3099<br>(-0.0333 – 0.6941)	0.0822<br>(-0.2422 – 0.4375)
Mycorrhizal models (ErM species)					
All	**0.4234**<br>**(0.2819 – 0.5671)**	0.1214<br>(-0.0432 – 0.3039)	**0.1694**<br>**(0.0195 – 0.3255)**	0.1301<br>(-0.0089 – 0.2817)	0.1014<br>(-0.0410 – 0.2478)
Sapling	-0.2275<br>(-0.7198 – 0.2581)	**0.9968**<br>**(0.2434 – 1.9454)**	-0.4523<br>(-0.7109 – 0.2991)	-0.1917<br>(-0.7109 – 0.2991)	0.3703<br>(-0.1562 – 0.9349)
Juvenile	0.1179<br>(-0.5717 – 0.8253)	0.9688<br>(-0.1801 – 2.3744)	0.2038<br>(-0.7445 – 1.2522)	-0.2268<br>(-0.9216 – 0.4265)	0.2121<br>(-0.7071 – 1.2389)
Adult	**0.3302**<br>**(0.1785 – 0.4851)**	0.0752<br>(-0.0979 – 0.2575)	**0.2287**<br>**(0.0678 – 0.3991)**	**0.2268**<br>**(0.0524 – 0.4171)**	0.0444<br>(-0.1217 – 0.2156)

Table S2. The parameter estimates and standard error (SE) for the relationship between the strength of neighborhood effects and sprouting ability (Sprout/main stem ratio), log-transformed abundance and their interaction terms for each species are shown. $\gamma_3 \sim \gamma_5$, extracted from species-level general models or mycorrhizal-specific models, represent neighborhood effects mediated by AM, EcM, and ErM trees, respectively. Significant results are highlighted in bold. P <0.05, *; P <0.01, **; P <0.001, ***.

Life stages	Parameter estimates (SE)								
	Sprout/main stem ratio			Log (Abundance)			Interaction		
	AM (γ_3)	EcM (γ_4)	ErM (γ_5)	AM (γ_3)	EcM (γ_4)	ErM (γ_5)	AM (γ_3)	EcM (γ_4)	ErM (γ_5)
General models									
All	0.0166<br>(0.0196)	0.0124<br>(0.0186)	-0.0160<br>(0.0245)	-0.0027<br>(0.0096)	0.0039<br>(0.0091)	-0.0041<br>(0.0120)	-0.0162<br>(0.0210)	-0.0037<br>(0.0199)	-0.0058<br>(0.0262)
Sapling	**0.0808 ****<br>**(0.0304)**	**0.0427 ***<br>**(0.0173)**	0.0326<br>(0.0531)	**0.0333 ***<br>**(0.0146)**	**0.0205 ***<br>**(0.0083)**	0.0109<br>(0.0255)	**-0.0896 ****<br>**(0.0325)**	**-0.0481 ***<br>**(0.0185)**	-0.0670<br>(0.0566)
Juvenile	-0.0147<br>(0.0535)	0.0074<br>(0.0433)	-0.0002<br>(0.0688)	-0.0024<br>(0.0288)	0.0101<br>(0.0234)	0.0085<br>(0.0371)	0.0133<br>(0.0585)	-0.0282<br>(0.0474)	-0.0208<br>(0.0754)
Adult	0.0106<br>(0.0118)	0.0015<br>(0.0109)	0.0007<br>(0.0176)	0.0083<br>(0.0056)	0.0070<br>(0.0052)	0.0062<br>(0.0084)	-0.0209<br>(0.0127)	-0.0067<br>(0.0118)	-0.0116<br>(0.0190)

Mycorrhizal models (AM species)									
All	0.0412	0.0070	0.0223	0.0123	0.0064	0.0183	**-0.0513 ***	-0.0115	-0.0548
	(0.0213)	(0.0099)	(0.0441)	(0.0092)	(0.0043)	(0.0189)	**(0.0226)**	(0.0105)	(0.0466)
Sapling	**0.1139 ***	0.0026	-0.0310	0.0369	0.0031	-0.0268	**-0.1072 ***	-0.0002	-0.0050
	**(0.0520)**	(0.0097)	(0.0935)	(0.0229)	(0.0043)	(0.0411)	**(0.0523)**	(0.0097)	(0.0939)
Juvenile	-0.0098	0.0077	0.0131	0.0145	0.0038	0.0108	-0.0254	-0.0358	-0.0321
	(0.0593)	(0.0482)	(0.0493)	(0.0359)	(0.0292)	(0.0298)	(0.0635)	(0.0517)	(0.0528)
Adult	0.0066	-0.0012	0.0015	0.0046	0.0015	0.0088	-0.0117	-0.0018	-0.0292
	(0.0092)	(0.0072)	(0.0037)	(0.0407)	(0.0037)	(0.0207)	(0.0099)	(0.0078)	(0.0439)
Mycorrhizal models (EcM species)									
All	-0.0539	0.0720	-0.2837	-0.0293	-0.0063	-0.0972	0.0695	-0.0577	0.3229
	(0.1136)	(0.1704)	(0.1716)	(0.0396)	(0.0594)	(0.0599)	(0.1278)	(0.1918)	(0.1932)
Sapling	0.0044	0.0438	0.0109	-0.0089	0.0112	0.0199	0.0087	-0.0193	0.0153
	(0.0746)	(0.1801)	(0.0926)	(0.0577)	(0.1393)	(0.0716)	(0.0804)	(0.1942)	(0.0998)
Juvenile	-0.0036	-0.2270	-1.4082	0.0722	-0.1075	-0.3160	-0.0288	0.2831	1.5107
	(0.4635)	(0.2600)	(1.0053)	(0.1633)	(0.0916)	(0.3543)	(0.5421)	(0.3041)	(1.1757)

Adult	-0.4086	-0.6705	-3.7463	-0.0185	-0.0232	-0.3925	0.4567	0.7804	3.8908
	(0.8110)	(0.5826)	(3.8818)	(0.1164)	(0.0836)	(0.5569)	(0.8573)	(0.6159)	(4.1035)
Mycorrhizal models (ErM species)									
All	-0.1718	0.0600	0.4336	-0.0351	0.1817	0.0542	0.1535	-0.0474	-0.3997
	(0.1816)	(0.7369)	(0.1907)	(0.0570)	(0.2314)	(0.0599)	(0.1868)	(0.7578)	(0.1962)
Sapling	-0.0462	1.6992	-0.5180	0.0482	0.7968	-0.0392	-0.0062	-1.9129	0.6809
	(0.0723)	(0.9528)	(0.0931)	(0.0323)	(0.4255)	(0.0416)	(0.0698)	(0.9207)	(0.0900)
Juvenile	1.5667	4.0460	-0.7675	-0.0548	0.4623	-0.6205	-1.3519	-3.9193	1.0744
	(3.5188)	(21.0261)	(3.3311)	(1.1316)	(6.7614)	(1.0712)	(3.6039)	(21.5346)	(3.4116)
Adult	-0.1630	0.0987	0.5211	0.0096	0.1973	0.0798	0.1169	-0.0513	-0.4836
	(0.4075)	(0.7808)	(0.3353)	(0.1232)	(0.2360)	(0.1013)	(0.4091)	(0.7839)	(0.3366)
